# Opportunities and challenges for introducing HPV testing for cervical cancer screening in sub-Saharan Africa

**DOI:** 10.1016/j.ypmed.2018.07.012

**Published:** 2018-09

**Authors:** Vivien Davis Tsu, Denise Njama-Meya, Jeanette Lim, Marjorie Murray, Silvia de Sanjose

**Affiliations:** a Reproductive Health, PATH, 2201 Westlake Avenue, Suite 200, Seattle, WA 98121, USA; b Reproductive Health, PATH, P.O. Box 7404, Kampala, Uganda

**Keywords:** Cervical cancer screening, HPV testing, Low-resource countries, East Africa

## Abstract

To protect women against cervical cancer, the World Health Organization recommends that women aged 30 to 49 years be screened with tests that detect human papillomavirus (HPV). If the countries that have the greatest burden of this disease—especially those in sub-Saharan Africa—are not to be left behind, we must understand the challenges they face and identify measures that can help them take full advantage now of innovations that are transforming screening services in wealthier countries. We reviewed policy documents and published literature related to Kenya, Tanzania, and Uganda, and met with key personnel from government and nongovernmental organizations.

National policy makers understand the value of HPV testing in terms of its superior sensitivity and the programmatic advantages that could result from using self-collected samples. However, while these countries have national cervical cancer prevention strategies, and some have national departments or units for cervical cancer prevention, screening is rare, funding scarce, and quality low. Age guidelines are not strictly followed, with scarce resources being used to screen many women younger than the recommended ages.

Published evidence of the benefits of HPV testing—including performance, safety, and cost-effectiveness—must be provided to ministry of health leaders, along with information on anticipated costs for training personnel, purchasing supplies, providing facility space, and maintaining test kits. Despite the obstacles, a joint effort on the part of global and national stakeholders to introduce molecular screening methods can bring better protection to the women who need it most.

## Highlights

• National policymakers in sub-Saharan Africa understand the value of HPV testing.
• Policy support for screening exists, but only limited financial commitment.
• Policymakers prefer locally-generated evidence on test performance.
• Processes for national recommendations and product registration are complex.
• Practical solutions can help reduce cost barriers and operational challenges.

The introduction of highly effective vaccines against human papillomavirus (HPV) has invigorated efforts to defeat cervical cancer (Drolet et al., 2015), a leading cause of cancer mortality in women in low-resource countries. Nearly nine in ten of the 260,000 annual deaths from cervical cancer occur in less-developed regions of the world (Ferlay et al., 2015). However, unvaccinated women can only be protected by screening for and treating precancerous lesions. The World Health Organization (WHO) has recommended that, where resources permit, women aged 30 to 49 years be screened with validated tests that detect HPV in cervical or vaginal samples (World Health Organization, 2013). These tests are more sensitive than visual inspection with acetic acid (VIA) or Pap smears, allow for longer screening intervals (minimum five years) (World Health Organization, 2013), and can be done with self-collected vaginal samples. The latest resource-stratified guidelines from the American Society of Clinical Oncology confirm HPV testing as the preferred screening option even in low-resource settings (Jeronimo et al., 2016a). But how feasible is this?

With sub-Saharan Africa having some of the highest rates of cervical cancer in the world, it is important to evaluate the opportunities for introducing HPV testing as the primary screening method in this region. To this end, we reviewed policy documents and published literature related to Kenya, Tanzania, and Uganda, and met with key personnel from government and nongovernmental organizations in Tanzania and Uganda—25 to 30 in each country. We reviewed current policies and practices, asked about evidence needed by governments to support changing policies, and explored the decision-making processes for changing screening policy.

Many experts in the region are well aware of the potential advantages of HPV testing as a primary screening strategy, but they face barriers to adopting it. If the countries that have the greatest need are not to be left behind as technologies advance, we must understand the challenges they face and identify key interventions to enable them to take full advantage now of the scientific and programmatic innovations that are transforming modern screening services in wealthier countries (Wentzensen et al., 2017). Below we briefly discuss current cervical cancer policies and programs in these countries, the processes for adopting HPV testing, and strategies to overcome barriers. While there are many additional issues that are important to scaling up HPV testing, such as which molecular test to choose, anticipated test prices, and clinical algorithms, this commentary focuses on important steps and programmatic concerns for national stakeholders interested in adopting this promising technology.

## 1 Current cervical cancer prevention programs

### 1.1 Policy support but limited financial commitment

All three countries have published national cervical cancer prevention strategies (Tanzania Ministry of Health and Social Welfare, 2011; Uganda Ministry of Health, 2010; Kenya Ministry of Public Health and Sanitation and Ministry of Medical Services, 2012). In addition, Tanzania has a cervical cancer unit in the Reproductive and Child Health Services department and a cervical cancer technical working group. In Uganda, prevention and treatment for all cancers is under the jurisdiction of the noncommunicable diseases program, and the Uganda Cancer Institute has the mandate to implement programs. In Kenya, implementation of the national screening program has been carried out in only a few sites and in individual projects rather than as a national program, and the programs are reportedly given low priority among policymakers and opinion leaders. In all three countries, the health management information systems collect data on only a few indicators.

Despite the existence of programs and cancer institutes, stakeholders in all countries noted that funding was scarce and that, for the most part, only donors and NGOs were implementing prevention and control strategies, and only in a very limited number of areas. Budget line items for cervical cancer are rare and are generally for policy activities rather than for implementation.

### 1.2 Age guidelines exist but are not strictly followed

WHO guidelines recommend cervical cancer screening for women aged 30 to 49 years (World Health Organization, 2013). In Tanzania, the ages are 30 to 50, and for Uganda and Kenya, the ages are 25 to 49 (Tanzania Ministry of Health and Social Welfare, 2011; Uganda Ministry of Health, 2010; Kenya Ministry of Public Health and Sanitation and Ministry of Medical Services, 2012). However, stakeholders reported that many women outside these ranges are screened, in part because screening is often offered in health facilities providing maternal care, where many women attending are younger than the recommended range. The recommended screening interval ranges from three years in Tanzania and Uganda to five years in Kenya, with shorter intervals for HIV-positive women.

### 1.3 Program coverage is low and quality varies

Where screening is done, VIA is the most common method. Challenges for screening programs include a low level of community awareness, inadequate skills among service providers, and lack of equipment and supplies—even though these are inexpensive for VIA. Screening coverage in Kenya was around 3.5% in 2003 (Institut Català d'Oncologia (ICO) Information Centre, 2016), although it is likely higher now because of considerable country effort to increase it; there is no data on coverage for Tanzania or Uganda. In Tanzania, women who have positive screening results are treated with cryotherapy, when available, but stakeholders reported gaps in service, inadequate resources, and a general lack of diagnostic and treatment referrals and services. In Uganda access to treatment for cervical precancer is limited, and patients referred to district or regional hospitals are often lost to follow-up.

## 2 Evidence needed for policy change

### 2.1 Comparing VIA and HPV screening

Many studies have evaluated the performance of different screening methods, and a recent systematic review and meta-analysis confirmed the consensus that HPV testing is more sensitive than VIA or Pap smears (Mustafa et al., 2016). In this review, 32 papers were considered eligible, of which 18 represented studies in low- and middle-income countries.

Programmatic advantages and disadvantages of VIA and HPV screening are widely recognized (Gupta et al., 2017) and were articulated by stakeholders in Tanzania, Uganda, and Kenya. Benefits of VIA include its low cost and immediate results, at the cost of poor sensitivity; while for HPV testing, benefits include self-collection of samples and a long screening interval but higher initial cost. Information on cost-effectiveness of screening methods and program requirements should be provided to ministries of health to aid decision-making.

### 2.2 Level of health system for HPV screening

Experts in the three countries differed in their assessment of what would be the best level of the health system for carrying out HPV screening. In Tanzania the consensus was for lower-level health facilities, while in Uganda, stakeholders proposed initiating the screening program at hospitals with laboratory capacity, then working in a cascade fashion to introduce it at sequentially lower levels of the health system. In Kenya, stakeholders noted that because of their decentralized system of government, each county would need to make its own decisions.

### 2.3 In-country research is desirable

Stakeholders agreed that governments would be most interested in research conducted in their own countries, and there have been studies of HPV testing in all three. With support from WHO and the International Agency for Research on Cancer, the Tanzania Ministry of Health (MOH) is conducting a pilot study to evaluate the feasibility and logistics of implementing HPV screening in existing VIA-based programs (World Health Organization, 2016). In Uganda, studies have assessed test performance and the acceptability of self-collection; the next step is to provide the results to the appropriate entities at the MOH (Moses et al., 2015; Jeronimo et al., 2014; Ogilvie et al., 2013). In Kenya, a pilot study is being conducted in collaboration with Moi Teaching and Referral Hospital, Kenya, and the University of Indiana, USA. The model includes a mobile clinic that offers same-day screen-and-treat services at one site while at another site, samples are collected and results provided within two to four weeks, depending on the rate of sample collection.

## 3 Decision-making process for adopting HPV testing as the primary screening method

### 3.1 Screening policy and guideline changes

Stakeholders noted that there is pressure to screen women younger than the WHO-recommended age of 30; to change this, the established evidence on the normal high rate of transient positive results—especially with HPV testing—among women under 30 must be conveyed. Guidelines for screening intervals also should be modified to reflect that less frequent screening is needed with HPV testing, and patient management flow charts must be revised.

### 3.2 Processes for recommendation and registration

Two processes will be needed to make HPV testing the primary screening method in these countries. First, the appropriate department at each MOH must recommend the change to the ministry leadership and provide published evidence of the method's benefits, including performance and cost-effectiveness studies. This should include countries/areas where HPV testing has been used, outcomes compared with other screening methods, and data on safety. Officials will need information on the costs anticipated for training providers and laboratory personnel, transporting specimens when necessary, purchasing supplies, providing facility space, and maintaining the test kits. Stakeholders suggested presenting information and evidence not only to high-level audiences but also to health care workers, managers, and women in communities, so these groups can advocate for change.

The second process is that of evaluation and registration of tests for commercial availability. Each manufacturer must apply for registration of its device to the appropriate agency, and these agencies may engage others to perform evaluations of the tests. However, devices recognized by WHO and bearing the European Union CE mark may receive expedited registration.

## 4 Looking ahead

National policy-makers working on cervical cancer already understand the value of HPV testing in terms of its superior sensitivity and the programmatic advantages that could result from using self-collected samples. The key concerns of stakeholders in three sub-Saharan African countries about introducing HPV screening included costs for purchasing tests and logistics of implementation. To a lesser extent, the desire for local evidence and the burden of changing national guidelines and policies were cited as barriers.

While cost-effectiveness analyses clearly show the advantage of HPV testing (Campos et al., 2015), they do not necessarily speak to the issue of affordability, and stakeholders remain skeptical that governments will adequately fund HPV testing.

Several practical actions can be taken to reduce costs of current HPV testing. First, countries can begin scaling up screening services, thereby increasing the volume of tests used, which will provide bargaining power for them as purchasers and a more reasonable market for manufacturers. Second, global agencies can support pooled procurement systems. One model for this is PATH's work with the Pan American Health Organization and the Council of Ministers of Health of Central America on pooled procurement options for Latin America. Third, national regulatory authorities could streamline processes of test registration (regional review or standardized registration templates) to reduce the barriers manufacturers now face, especially in small countries where the low volume of sales discourages investment.

To address the programmatic challenges of sample collection and transport, timely return of test results, and follow-up of test-positive women, countries must make adjustments in their health systems. Fortunately, there are good models from other health areas (e.g., transport of tuberculosis samples and follow-up of those who test positive) as well as from other countries that are already switching to HPV testing, including use of self-collected samples, such as Argentina and Central American countries (Arrossi et al., 2017; Jeronimo et al., 2016b). Nongovernmental organizations in East Africa have begun to explore ways to overcome these operational barriers using HPV tests in small pilots in both clinic and mobile settings (Makula, 2017). Global agencies could facilitate the sharing of lessons on how to manage these logistical challenges.

While researchers working to improve HPV testing assert that less expensive, easier-to-use break-through technologies are on the way to address these difficulties, commercial and regulatory approvals will take time. Rather than waiting, we can take actions now to protect women and to build the program infrastructure into which newer technologies can be inserted when ready. In 20 years, the first generations of vaccinated girls will reach screening age and greatly reduce the urgency and total costs of screening, but we must consider the needs of women who could benefit now from tests that are already available. Technologies will continue to advance, but they will always rely on solid programs and policies in robust health systems for successful deployment.

The recent call from WHO for the elimination of cervical cancer (Ghebreyesus, 2018) will strengthen the resolve of countries and their global partners and should generate more support for effective and affordable screening. Our landscaping exercise demonstrates that despite the obstacles they face in adopting HPV testing for screening, sub-Saharan countries have the capability—using the practical measures outlined above and with appropriate global support—to move ahead to protect the women of Africa from cervical cancer now.

## References

[bb0005] Arrossi S., Paolino M., Thouyaret L., Laudi R., Campanera A. (2017). Evaluation of scaling-up of HPV self-collection offered by community health workers at home visits to increase screening among socially vulnerable under-screened women in Jujuy Province, Argentina. Implement. Sci..

[bb0010] Campos N.G., Castle P.E., Wright T.C., Kim J.J. (2015). Cervical cancer screening in low-resource settings: a cost-effectiveness framework for valuing tradeoffs between test performance and program coverage. Int. J. Cancer.

[bb0015] Drolet M., Bénard É., Boily M.C., Ali H., Baandrup L., Bauer H., Brisson M. (2015). Population-level impact and herd effects following human papillomavirus vaccination programmes: a systematic review and meta-analysis. Lancet Infect. Dis..

[bb0020] Ferlay J., Soerjomataram I., Dikshit R., Eser S., Mathers C., Rebelo M., Bray F. (2015). Cancer incidence and mortality worldwide: sources, methods and major patterns in GLOBOCAN 2012. Int. J. Cancer.

[bb0025] Ghebreyesus T.A. (2018). Cervical Cancer: An NCD We Can Overcome. http://www.who.int/reproductivehealth/DG_Call-to-Action.pdf.

[bb0030] Gupta R., Gupta S., Mehrotra R., Sodhani P. (2017). Cervical cancer screening in resource-constrained countries: current status and future decisions. Asian Pac. J. Cancer Prev..

[bb0035] Institut Català d'Oncologia (ICO) Information Centre (2016). Human Papillomavirus and Related Diseases Report: Kenya. http://www.hpvcentre.net/statistics/reports/KEN.pdf.

[bb0040] Jeronimo J., Bansil P., Lim J., Peck R., Paul P., Amador J.J., Poli U.R. (2014). A multicountry evaluation of careHPV testing, visual inspection with acetic acid, and Papanicolaou testing for the detection of cervical cancer. Int. J. Gynecol. Cancer.

[bb0045] Jeronimo J., Castle P.E., Temin S., Denny L., Vandana G.V., Kim J.J., Shastri S. (2016). Secondary prevention of cervical cancer: ASCO resource-stratified clinical practice guideline. J. Glob. Oncol..

[bb0050] Jeronimo J., Holme F., Slavkovsky R., Camel C. (2016). Implementation of HPV testing in Latin America. J. Clin. Virol..

[bb0055] Kenya Ministry of Public Health and Sanitation and Ministry of Medical Services (2012). National Cervical Cancer Prevention Program: Strategic Plan 2012–2015. https://www.k4health.org/toolkits/kenya-health/national-cervical-cancer-prevention-program-strategic-plan-2012-2015.

[bb0060] Makula J.A. (2017). Implementing a single-visit approach with careHPV in Tanzania. IFCPC 2017 World Congress for Cervical Pathology and Colposcopy, April 4–7.

[bb0065] Moses E., Pedersen H.N., Mitchell S.M., Sekikubo M., Mwesigwa D., Singer J., Ogilvie G.S. (2015). Uptake of community-based, self-collected HPV testing vs. visual inspection with acetic acid for cervical cancer screening in Kampala, Uganda: preliminary results of a randomised controlled trial. Tropical Med. Int. Health.

[bb0070] Mustafa R.A., Santesso N., Khatib R., Mustafa A.A., Wiercioch W., Kehar R., Schünemann H.J. (2016). Systematic reviews and meta-analyses of the accuracy of HPV tests, visual inspection with acetic acid, cytology, and colposcopy. Int. J. Gynecol. Obstet..

[bb0075] Ogilvie G.S., Mitchell S., Sekikubo M., Biryabarema C., Byamugisha J., Jeronimo J., Money D.M. (2013). Results of a community-based cervical cancer screening pilot project using human papillomavirus self-sampling in Kampala, Uganda. Int. J. Gynecol. Obstet..

[bb0080] Tanzania Ministry of Health and Social Welfare (2011). Tanzania Service Delivery Guidelines for Cervical Cancer Prevention and Control 2011–2015.

[bb0085] Uganda Ministry of Health (2010). Strategic Plan for Cervical Cancer Prevention and Control in Uganda 2010–2014. http://www.rho.org/files/PATH_Uganda_cxca_strat_plan_2010-2014.pdf.

[bb0090] Wentzensen N., Arbyn M., Berkhof J., Bower M., Canfell K., Einstein M., Franceschi S. (2017). Eurogin 2016 roadmap: how HPV knowledge is changing screening practice. Int. J. Cancer.

[bb0095] World Health Organization (2013). WHO Guidelines for Screening and Treatment of Precancerous Lesions for Cervical Cancer Prevention. http://apps.who.int/iris/bitstream/10665/94830/1/9789241548694_eng.pdf?ua=1.

[bb0100] World Health Organization (2016). AISHA: An Implementation Study on Rapid HPV Testing in Tanzania. http://www.who.int/reproductivehealth/projects/HRX18_AISHA_UWHORTanzania.pdf.

